# Clippings from Correspondence

**Published:** 1868-08-15

**Authors:** 


### *Clippings from Correspondence.*

*Position in treatment of Sore Nipples.*—A. S. V. MANSFELDE, M.D., of this city, in a communication to us upon this subject, says:

"It will be noticed that the ulcers caused by the pressure of the gums or teeth of the infant, cover not one-half at once, but the two opposite quarters of the nipple of each mammæ; that is owing to the '*position*' of the child while nursing. It lays in the arms of the mother, and is changed from the right to the left, while nursing upon the left or right breast. The infant, as you know, reposes upon the right side, when using the left pap. mam. Now, what I do and with the best result, is, simply directing the nurse to keep the child upon the same side when using the right breast. To accomplish this, I order a chair to be placed by the right side of the mother, covered with a pillow, high enough to reach to the nipple. Upon this the infant is placed, and nursing now, it will touch with its semi-circle of teeth or gums, the opposite two quarters of the nipple, thus giving chance for the remaining parts to heal. But changed in time none of them will ever get diseased, being cared for additionally as directed by authors upon this subject. Consequently our mothers will no longer suffer such agonizing pain, and the doctor will no longer be troubled with the cure of '*sore nipples.*'"

*Pernicious Effects of the Bromide of Potassium.*—Dr. N. TEAL, of Kendallville, Indiana, says:

"In your issue of July 1st inst., you ask for observations upon the '*pernicious* effects of the too long continued and excessive use of *Bromide of Potossium*,' and in compliance I beg leave to submit a single case:

"Mrs. ——, aged 70, was put upon—

℞. *Bromide of Potassium* . . . ʒj.

*Aq. Dist.* . . . . . . . ℥j.

M. S. One-half teaspoonful every six to eight hours daily.

"The above was continued for two weeks when the tongue, which had been much furred for several months previous, suddenly cleaned off and became quite sore. On examination I found the fauces and sides and roof of the mouth red and tender. Water taken into the mouth, if cold, gave pain, and when drank it 'hurt the stomach.' There was tenesmus and a smarting sensation after voiding the fæces. Judging from what I could see and hear of this case, I concluded the whole alimentary canal was more or less irritated, as I believe, from the continued use of the *Bromide.* Saw my patient a few days after she began to complain from the soreness of her mouth and tongue; discontinued the use of the *Bromide*, ordered emolient drinks, astringent gargles, etc. In a day or two the mouth and tongue were healed, and all went on well."

